# A promising routine to fabricate GeSi nanowires via self-assembly on miscut Si (001) substrates

**DOI:** 10.1186/1556-276X-6-322

**Published:** 2011-04-11

**Authors:** Zhenyang Zhong, Hua Gong, Yingjie Ma, Yongliang Fan, Zuimin Jiang

**Affiliations:** 1 State Key Laboratory of Surface Physics and Department of Physics, Fudan University, Handan Str. 220, Shanghai 200433, China

## Abstract

Very small and compactly arranged GeSi nanowires could self-assembled on vicinal Si (001) substrates with ~8° off toward ⟨110⟩ during Ge deposition. The nanowires were all oriented along the miscut direction. The small ration of height over width of the nanowire indicated that the nanowires were bordered partly with {1 0 5} facets. These self-assembled small nanowires were remarkably influenced by the growth conditions and the miscut angle of substrates in comparison with large dome-like islands obtained after sufficient Ge deposition. These results proposed that the formation of the nanowire was energetically driven under growth kinetic assistance. Three-dimensionally self-assembled GeSi nanowires were first realized via multilayer Ge growth separated with Si spacers. These GeSi nanowires were readily embedded in Si matrix and compatible with the sophisticated Si technology, which suggested a feasible strategy to fabricate nanowires for fundamental studies and a wide variety of applications.

**PACS:** 81.07.Gf, 81.16.Dn, 68.65.-k, 68.37.Ps

## Introduction

Semiconductor nanowires have attracted enormous attention as building blocks for nanoscale electronics [1], photonics [2], energy conversion and storage [3,4], thermoelectrics [5], and interfacing with living cells [6], thanks to their unique electronic, optical, and phonon properties [7]. In particular, Si-based nanowires were broadly investigated due to their compatibility with the sophisticated Si technology [1,3-6]. The nanowires can be obtained by top-down methods [8]. They can also be fabricated by bottom-up methods, including via vapor-liquid-solid (VLS) process [9], via solution-liquid-solid (SLS) process [10], through vapor phase conversion and transport processes [11], etc. However, it is always a big challenge to apply the nanowires obtained by those methods in electronic devices, such as recently proposed nanowire transistors without junctions [12], and the subsequent integration. The lateral nanowires directly realized on a substrate are more promising candidates for the fabrication of nanowire-based devices and the subsequent integration. Enormous effort has been devoted to find a routine to realize laterally arranged nanowires [13]. Recently, it was found that lateral InGaAs nanowires could be self-assembled on GaAs (001) substrates by multilayer growth of InGaAs/GaAs [14]. Depending on In content, lateral InGaAs nanowires could also be realized on GaAs (311)A substrates [15]. On very-high-index Si (*hkl*) substrates, self-assembled GeSi nanowires could be obtained in template grooves composed of corrugated hill-valley structure [16]. On miscut Si (001) substrates, GeSi nanostructures of various shapes could be obtained [17-21]. Especially, self-assembled Ge or GeSi nanowires could be fabricated by Stranski-Krastonov growth mode [17,20,21] on Si (113) substrates or on Si (001) substrates with ~8° off toward ⟨110⟩. On the other hand, these laterally self-assembled nanowires were always not uniform. Some self-assembled nanowires waggled or bifurcated. Such non-uniformity or waggling or bifurcating of the nanowires might give rise to the localization of carriers, which has been used to explain the small polarization anisotropy generally associated with nanowires [14]. Those self-assembled nanowires were believed to be strain-driven. However, the inherent mechanism, particularly the effect of the growth conditions and/or the surface microstructure, on the formation of self-assembled nanowires was still not so clear. For investigation of fundamental properties and device applications of nanowires, uniformed and even size-controlled nanowires were always required. To optimize growth conditions and/or surface microstructure for the required GeSi nanowires via self-assembly, it is demanded to further study Ge/GeSi nanowires growth on miscut Si substrates.

In this letter, we systematically investigated effects of the growth temperature and the miscut angle of substrates on self-assembled GeSi nanostructures on miscut Si (001) substrates during Ge deposition. It was found that very small and highly dense Ge nanowires can be readily self-assembled on Si (001) substrates with ~8° off toward ⟨110⟩. The nanowires oriented along the miscut direction. The small ratio of height over width of the nanowire indicated that the sidewall of the nanowire is partly composed of {1 0 5} facets. Moreover, with increasing growth temperature, the height of nanowires tended to decrease while the width of nanowires does not monotonically change. The self-assembled nanowires were also found to be sensitively dependent on the miscut angle. The growth mechanism of nanowires on miscut Si substrates was qualitatively discussed in terms of growth kinetics and energetics. By multilayer growth separated with Si spacers, three-dimensionally self-assembled GeSi nanowires were first obtained on miscut substrates, which provided with an additional way to control the density of nanowires or the distance between the nanowires. The present method provides a feasible routine to fabricate desired GeSi nanowires, which could be embedded in Si matrix and compatible with the sophisticated Si technology. These self-assembled GeSi nanowires could promote the exploration of the properties and the applications of nanowires.

## Experimental procedure

The samples were grown by molecular beam epitaxy (MBE) in a Riber Eva-32. Most samples were grown on Si (001) substrates with ~8° off toward ⟨110⟩, which is also called as (1 1 10) substrates [21]. Some samples were grown on Si (001) substrates with ~2°, ~4°, and ~10° off toward ⟨110⟩ to systematically study the formation of nanostructures on vicinal substrates. All substrates were cleaned using Shiraki method followed with HF treatment to form hydrogen terminated surface. After a thermal desorption, a ~100-nm-thick Si buffer layer was grown at a rate of 0.5 Å/s to obtain smooth and clean surface. To avoid kinetic step-bunching during Si buffer layer growth on vicinal Si (001) substrates [22], the first 50 nm Si was grown at 550°C, and another 50 nm Si was grown at 580°C. The surface of the miscut Si (001) substrate after Si buffer layer growth was very smooth without pronounced step-bunching observed by atomic force microscopy (AFM) (not shown). Ge were then deposited on the vicinal Si (001) substrates at different temperatures from 530 to 600°C at a growth rate of 0.08 Å/s. Considering the Ge-Si intermixing during Ge deposition, we believed that the obtained nanostructures were GeSi alloy. For the multilayer samples, 0.8 nm Ge was deposited at 530°C in each layer, which was separated by ~10 nm Si spacer with ramping substrate temperature from 500 to 530°C to suppress Ge-Si intermixing during spacer growth. The surface morphologies of the samples were investigated by AFM (Veeco DI Multimode V SPM) using tapping mode.

## Results and discussion

**Figure 1:**
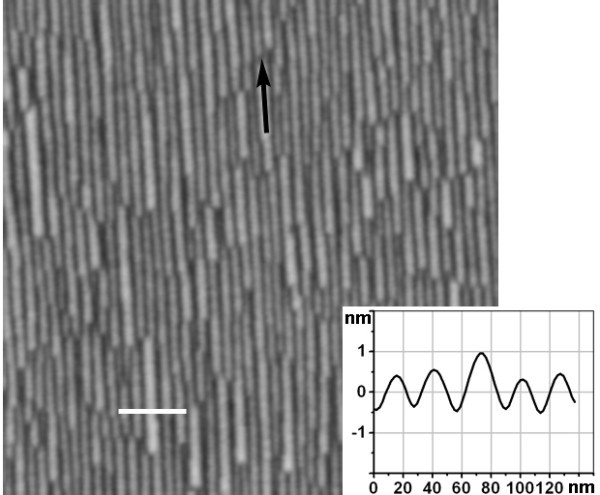
**AFM image (1 × 1 μm^2^) of GeSi nanowires after 0.8 nm Ge deposition on a vicinal Si (001) substrate with ~8° off toward ⟨110⟩ at 560°C**. The black arrow indicates the miscut direction. The inset shows the height profile along the white line in the figure.

Figure 1 showed the surface morphology after 0.8 nm Ge deposition on a Si (001) substrate with ~8° off toward ⟨110⟩ at 560°C. The compact GeSi nanowires were clearly demonstrated. The nanowires were found to be oriented along the miscut direction of ⟨110⟩, as denoted by a black arrow in the figure. This result was consistent with previous ones [20,21]. The height profile along the white line in the figure was shown in the inset of figure, which clearly exhibited the height and the width of the nanowires. The statistical analyses of the height and the width of the GeSi nanowires were 0.84 nm (± 0.28 nm) and 25.2 nm (± 6.41 nm), respectively. The small ratio of height over width of the nanowires demonstrated that these nanowires were much smaller than the GeSi nanowires of lower Ge composition [21]. This is mainly attributed to the high Ge content in the present nanowires with a large misfit strain. It has been found that the sidewalls of such nanowires were mainly composed of {1 0 5} facets [20,21]. However, in our cases, the ratio of height over width of the present nanowires was considerably smaller than that (~0.07) of the nanowires only composed of {1 0 5} facets. Considering {1 0 5} to be energetically favorable facets [23], we proposed that the sidewalls of the present nanowires were partly composed of {1 0 5} facets and others, which can not be distinguished due to the limitation of the resolution of AFM. This different result was attributed to the growth kinetic limitation at the low growth temperature and the high growth rate. Furthermore, the formation of such small nanowires was more energetically favorable than the layer-by-layer growth during Ge deposition on vicinal substrates after a critical thickness [20]. These self-assembled GeSi nanowires on miscut Si (001) substrates was different from that on very-high-index Si (*hkl*) substrates, where the GeSi nanowires were along the grooves composed of hill-and-valley structure [16].

**Figure 2:**
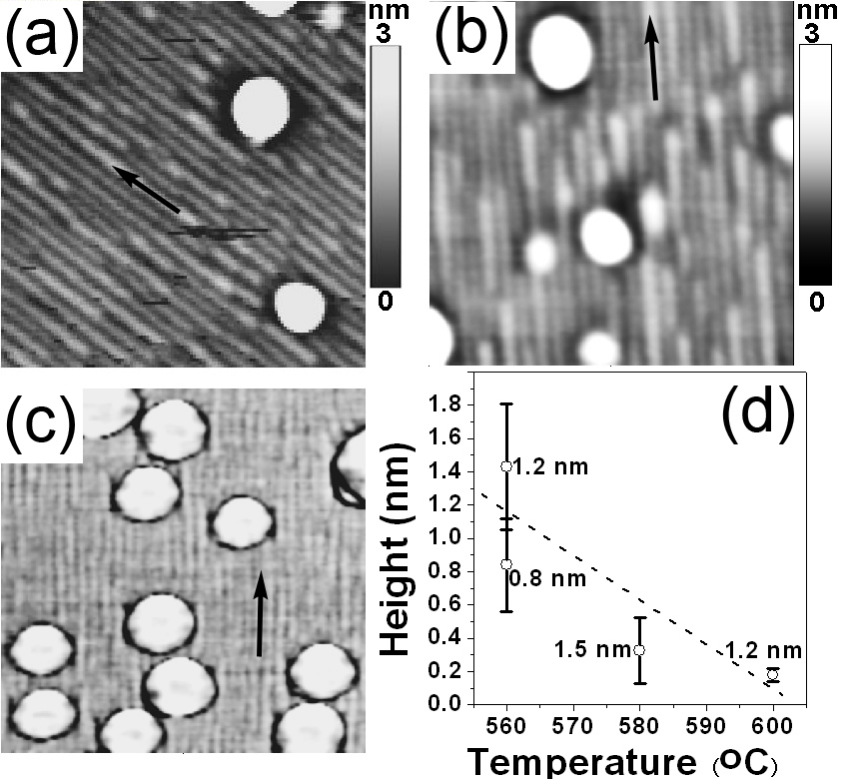
**AFM image (0.5 × 0.5 μm**^**2**^**) of the surface morphology after**. **(a) **1.2 nm Ge at 560°C, **(b) **1.5 nm Ge at 580°C, (c) 1.2 nm Ge at 600°C (phase image), on vicinal Si (001) substrates with ~8° off toward ⟨110⟩, **(d) **height of nanowires vs growth temperature. The black arrows in **(a)**, **(b)**, and **(c) **denote the miscut direction of the substrates. The dashed line in **(d) **is for eye-guide. The numbers in **(d) **are the corresponding amount of nominal Ge deposition.

It is well known that on normal Si (001) substrates dome-like GeSi islands can be obtained with sufficient Ge deposition [24], and the growth conditions affect the formation of the islands [25]. We found that the nanostructures grown on vicinal Si (001) substrates also depended on the amount of deposited Ge and the growth conditions, as shown in Figure 2. With sufficient Ge deposition, dome-like islands appeared in addition to the nanowires, as shown in Figure 2a-c. Such dome-like islands on vicinal Si (001) substrates were nearly the same as those on normal Si (001) substrates. The nanowires were still obtained and covered most of the surface area in these samples. The orientation of the nanowire, denoted by black arrows in Figure 2a-c, was all along the miscut direction. More interestingly, statistical analyses of the height of the nanowires of these samples demonstrated that the height of the nanowires tended to decrease with increasing growth temperature, as shown in Figure 2d. While the width of the nanowire was not so much different. Such a tendency of the height was related to the temperature-dependent Ge-Si intermixing [26]. The misfit strain due to the lattice mismatch between the epilayer and the substrate can be relaxed by three-dimensional (3D) growth and/or by alloying due to intermixing. At a low growth temperature, Ge-Si intermixing can be considerably reduced. Therefore the misfit strain was mainly relaxed by the 3D growth, which gave rise to the formation of nanowires with a large height. Whereas, at a high growth temperature, strong Ge-Si intermixing can efficiently relax the misfit strain. As a result, the finally formed nanowires had a small height. This result suggested that the formation of the nanowires was energetically driven. On the other hand, to obtain pronounced nanowires, the growth temperature should not be too high.

**Figure 3:**
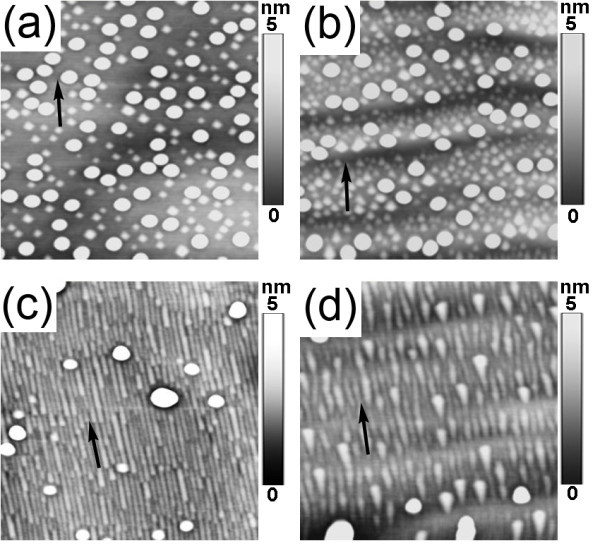
**AFM image (1 × 1 μm^2^) of the surface morphology after 1.1 nm Ge deposition at 560°C on vicinal Si (001) substrates with**. **(a) **2°, **(b) **4°, **(c) **8°, **(d) **10° off toward ⟨110⟩. The black arrows denote the miscut direction.

We also found that self-assembled GeSi nanowires on vicinal Si (001) substrates were sensitively associated with the miscut angle. Figure 3 showed the surface morphologies after ~1.1 nm Ge deposition at 560°C on vicinal Si (001) substrates with 2°, 4°, 8° and 10° off toward ⟨110⟩. On normal Si (001) substrates, both pyramid-like and dome-like GeSi islands can be realized with sufficient Ge deposition. In our cases, due to sufficient Ge deposition, dome-like GeSi islands were also obtained in all samples, which were nearly not affected by the miscut angle. However, the general pyramid-like islands with square base were essentially transferred into nanowires on the vicinal Si (001) substrates with 8° off, which were along the miscut direction denoted by a black arrow in Figure 3c. On the other vicinal Si (001) substrates, the general pyramid-like islands with square base were transformed into asymmetrical pyramid-like islands, which was elongated along the miscut direction denoted by black arrows in Figure 3b-d. In addition, the larger miscut angle of the substrate, the more pronounced elongation of the islands along the miscut direction. These results indicated that the step structures on miscut substrates can considerably affect the small nanostructures rather than the big ones such as big dome-like islands. An important reason is that the step at interface between the small nanostructures and the substrate played an important role in the strain relaxation; whereas it can be neglected for large dome-like islands.

**Figure 4:**
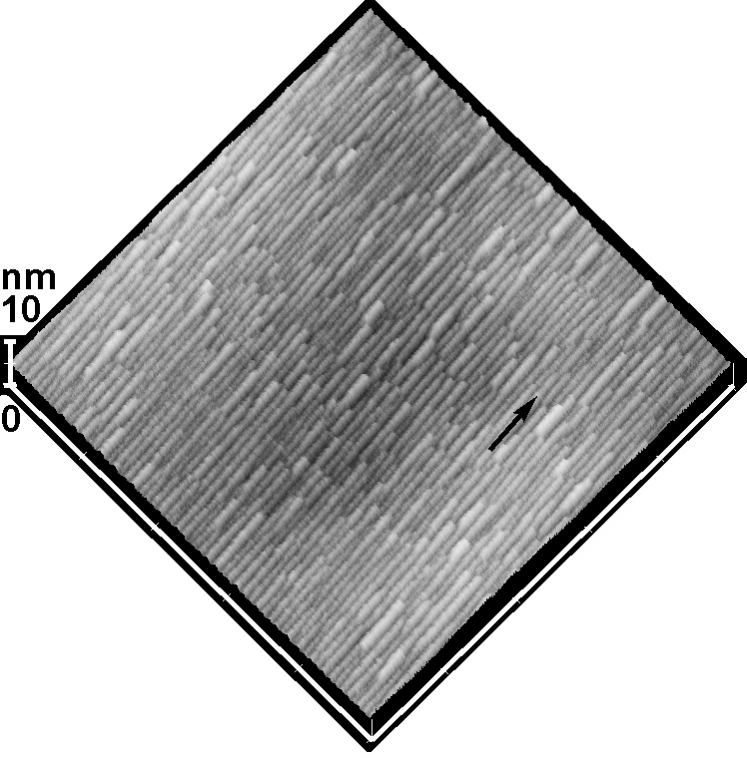
**AFM image (1 × 1 μm^2^) of the surface morphology after 10th layer of Ge growth on a vicinal Si (001) substrate with ~8° off toward ⟨110⟩**. The black arrow denotes the miscut direction.

Multilayer GeSi nanowires separated with thin Si spacers were realized on vicinal Si (001) substrates with 8° off toward ⟨110⟩. Figure 4 showed the surface morphology after 10th layer of Ge growth. The GeSi nanowires were clearly demonstrated. The orientation of the nanowire, denoted as a black arrow in Figure 4, was also along the miscut direction. The size of the nanowires was not so much different from that on the single layer sample. Considering the small height (< 1 nm) of the nanowires and the relatively thicker Si spacer (10 nm), it is reasonable to believe that the surface after each Si spacer growth was still flat (1 1 10), and the strain distribution on the spacer surface due to the buried nanowires could be neglected. Taking the low growth temperature into account, the segregation of Ge can be suppressed. In other words, GeSi nanowires were independently self-assembled in each layer during Ge deposition. As a result, the GeSi nanowires in each layer of the multilayer sample could be not so much different. Analogue to the multilayer GeSi islands growth [27], vertically aligned GeSi nanowires were expected by modulating the amount of Ge deposition and the thickness of Si spacer layer.

The studies on the initial evolution of Ge ripple structures on Si (1 1 10) have suggested that the step-flow process led to the formation of ripple structures [20]. Based on our results, we proposed that the step-flow process and the formation of the GeSi nanowires were energetically driven. It is not necessary that the nanowires were only bordered by two {1 0 5} facets, as was previously reported [20,21]. The formation of the nanowires partly bordered by {1 0 5} faces can efficiently relax the misfit strain perpendicular the nanowires. The misfit strain along the nanowires, to some degree, can be relaxed by D_B _steps on the Si (1 1 10) surface, which are perpendicular to the nanowires [20]. The formation of the nanowires was also kinetically assisted. By optimizing growth conditions, small nanowires bordered by only {1 0 5} facets were expected, which might result in more uniform and ordered GeSi nanowires. Such self-assembled GeSi nanowires could be readily embedded in Si matrix. This means that the characterization and the device fabrication of these nanowires can be easily done using sophisticated Si technology. Therefore, these self-assembled GeSi nanowires could be the promising candidate for exploration of the unique properties and the novel device applications of nanowires.

## Conclusions

In summary, GeSi nanowires were achieved by self-assembly on miscut Si (001) substrates with 8° off toward ⟨110⟩. The nanowires were along miscut directions and compactly arranged. They were partly bordered by {1 0 5} facets due to growth kinetic limitation. The formation of the nanowire was energetically driven and affected by the growth conditions and the miscut angle of substrates. The growth mechanism of GeSi nanowires on vicinal Si (001) substrates were further clarified, which would help to optimize the growth conditions to obtained desired nanowires. Multilayer GeSi nanowires separated with Si spacers were also realized. The present results demonstrated a feasible way to fabricate lateral GeSi nanwires, which could serve as a prototype model in investigation of the fundamental properties and the novel applications of nanowires.

## Abbreviations

AFM: atomic force microscopy; MBE: molecular beam epitaxy; SLS: solution-liquid-solid; 3D: three-dimensional; VLS: vapor-liquid-solid.

## Competing interests

The authors declare that they have no competing interests.

## Authors' contributions

ZZ coordinated the interpretation of the results and wrote the manuscript. HG grew the samples by MBE and helped with the AFM measurement. YM participated the sample growth and the AFM measurement. YF participated in the sample growth. ZJ participated in the design of the study.
